# A randomized controlled pilot trial to assess the effectiveness of a specially formulated food supplement and pelvic floor muscle training in women with stress-predominant urinary incontinence

**DOI:** 10.1186/s12905-023-02476-z

**Published:** 2023-06-20

**Authors:** Peter Takacs, Krisztina Pákozdy, Erzsébet Koroknai, Balázs Erdődi, Zoárd Krasznai, Bence Kozma

**Affiliations:** 1 Department of Obstetrics and Gynecology, Division of Female Pelvic Medicine and Reconstructive Surgery, Eastern Virginia Medical School, 825 Fairfax Avenue, Suite 526, Norfolk, VG 23507-2007 USA; 2 Present Address: Faculty of Medicine, Department of Obstetrics and Gynecology, University of Debrecen, Pf 400, Debrecen, 4002 Hungary

**Keywords:** Urinary incontinence, Pelvic floor, Creatine, Zinc, Leucine

## Abstract

**Background:**

Pelvic floor muscle training (PFMT) is the first-line treatment approach for stress urinary incontinence. Creatine and leucine have been shown to improve muscle function. Our aim was to assess the effectiveness of a food supplement and PFMT in women with stress-predominant urinary incontinence.

**Methods:**

Women with stress-predominant urinary incontinence were randomized in 1:1 ratio to receive daily oral supplementation for six weeks with either a food supplement (treatment group) or placebo (control group). Both groups were instructed to perform standardized daily PFMT. The primary outcome was the Urogenital Distress Inventory Short Form (UDI-6) score. Secondary outcomes were the Incontinence Impact Questionnaire (IIQ-7) score, Patient’s Global Impression of Severity (PGI‐S), and Biomechanical Integrity score (BI-score) measured by Vaginal Tactile Imager. To have a power of 80% and a significance level of 5% to detect a decrease of 16 points in the UDI-6 score, a sample size of 32 was needed, with 16 patients in each arm of our trial.

**Results:**

Sixteen women in the control group and sixteen in the treatment group completed the trial. Between-group analysis revealed no significant differences between the control and treatment group except for mean change (delta) in vaginal squeeze pressure [(cmH2O, mean ± SD), 5 ± 12 vs. 15 ± 15, P = 0.04] and mean change (delta) in PGI-S score [(mean ± SD), -0.2 ± 0.9 vs. -0.8 ± 0.8, P = 0.04]. Within-group analysis showed that UDI-6 and IIQ-7 scores improved significantly from baseline to six weeks in the treatment group but not in the control group [UDI-6 score (mean ± SD) 45 ± 21 vs. 29 ± 21, P = 0.02; 43 ± 18 vs. 33 ± 26, P = 0.22] [IIQ-7 score (mean ± SD) 50 ± 30 vs. 30 ± 21, P = 0.01; 48 ± 23 vs.40 ± 28, P = 0.36]. PGI-S scores only improved in the treatment group from baseline to six weeks after treatment [PGI-S score (mean ± SD) 3.1 ± 0.8 vs. 2.3 ± 0.8, P = 0.0001]. BI-score, on average, improved significantly in the treatment and control group as well [SD unit, mean, from − 1.06 to -0.58, P = 0.001; from − 0.66 to -0.42, P = 0.04].

**Conclusions:**

Women with stress-predominant urinary incontinence receiving a specially formulated supplement in addition to daily PFMT for six weeks had significantly improved urinary symptoms (decrease in UDI-6 score and IIQ-7) and BI-score compared to their baseline.

**Trial registration:**

ClinicalTrials.gov Identifier: NCT05358769. 27/04/2022.

## Background

Stress urinary incontinence (SUI) is the complaint of involuntary leakage on effort or exertion or on sneezing or coughing, while mixed urinary incontinence is the complaint of involuntary leakage associated with urgency and also with exertion, effort, sneezing, or coughing [1]. Mixed urinary incontinence (MUI) is regarded as stress-predominant when stress incontinence episodes dominate [2]. Approximately 50% of women will experience some form of urinary incontinence (UI) in their lifetime, as prevalence and age are positively correlated [3]. UI is a significant economic burden, with annual cost estimates of $19.5 billion or more [4].

Despite advancements in the management of SUI, conservative treatment is recommended by most professional guidelines. The European Association of Urology (EAU) recommends that providers should offer supervised intensive pelvic floor muscle training (PFMT), lasting at least three months, as first-line therapy to all women with SUI or MUI (including elderly women) and to ensure that PFMT programs are as intensive as possible [5]. The American College of Obstetricians and Gynecologists (ACOG) Practice Bulletin on urinary incontinence states that behavioral modification and pelvic floor muscle exercises improve symptoms of urinary incontinence and may be recommended as an initial, noninvasive treatment in any woman [6]. Although PFMT is recommended as the first-line treatment for UI by most professional organizations, there is no consensus on the number, duration, and intensity of the exercises. Previous publications have described the rationale for adequate PFMT in Effects of Surgical Treatment Enhanced with Exercise for Mixed Urinary Incontinence (ESTEEM) trial [7–9]. In ESTEEM exercise program of 45 PFM contractions per day was considered adequate exercise volume to improve muscle fitness [7, 8]. This specific PFMT volume has been shown to be manageable with regard to exercise adherence and improved urinary incontinence symptoms [10, 11]. A recent Cochrane systemic review compared PFMT with no treatment or inactive control treatment and found that women with SUI in the PFMT groups were eight times more likely to report cure [12].

Creatine is a non-essential nitrogenous organic acid, the primary constituent of phosphocreatine, used to regenerate ATP within the cell [13–15]. Depending on muscle mass, the body must replenish about 1–3 g of creatine daily to maintain normal creatine stores. Nearly half of the daily need for creatine is obtained from the diet [16, 17].

The European Food Safety Authority (EFSA) has approved several health claims for creatine, including that daily creatine consumption can enhance the effect of resistance training on muscle strength in adults over the age of 55 and that creatine increases physical performance in successive bursts of short-term, high-intensity exercise [18]. Since pelvic floor muscle exercise has been shown to improve the symptoms of stress incontinence, and creatine supplementation may augment muscle training, we considered that women with stress urinary incontinence might benefit from supplementation with creatine while practicing pelvic floor muscle exercises.

Besides creatine, leucine has been extensively studied regarding improving muscle function. Leucine is an essential amino acid capable of stimulating the synthesis of muscle proteins. It enhances regeneration and prevents muscle tissue from degradation [19]. In addition, several research studies have shown leucine’s beneficial role in enhancing muscle protein synthesis [19–21]. For example, postpartum women receiving a specially formulated leucine-containing postpartum recovery supplement had improved pelvic floor recovery after vaginal delivery [22].

Our pilot study aimed to investigate the impact of a specially formulated creatine-leucine-zinc-calcium-magnesium-containing supplement in addition to regular pelvic floor muscle exercise on urinary incontinence symptoms and improvement in pelvic floor muscle strength. This supplement was designed to enhance the effect of resistance training on muscle strength and to increase physical performance in successive bursts of short-term, high-intensity exercise, which could help with stress-induced urinary incontinence symptoms. We hypothesized that the combination of daily pelvic floor exercise and supplementation with a special dietary supplement would improve stress urinary incontinence symptoms, partly secondary to increased pelvic floor muscle strength.

## Methods

We have conducted a randomized, double-blind, placebo-controlled pilot trial with a six-week follow-up period at an outpatient medical center between 4/1/22 − 9/1/22. The trial was registered at ClinicalTrials.gov (Identifier: NCT05358769), and IRB approved by the Scientific and Research Ethics Committee of Hungary: 2876-13/2022/EÜIG.

The randomization was done in a 1:1 ratio and generated by SAS (SAS Institute, Cary, NC, USA) version 9.4. We have enrolled women with stress or stress-predominant urinary incontinence [based on the Medical, Epidemiologic, and Social Aspects of Aging (MESA) questionnaire] [23, 24]. Women who indicated stress UI or stress-dominant mixed UI (stress percent score more than urge percent score) was eligible for inclusion.

Exclusion criteria were pregnancy or less than 12 months postpartum; more than three vaginal deliveries or any prior operative delivery; self-reported symptoms of pelvic organ prolapse or POP-Q stage > 2; history of supervised PFMT within 12 months; and current medications for UI or surgical treatment for UI; known zinc or copper deficiency or sensitivity; collagen or connective tissue disease.

After enrollment, women were randomized to receive the specially formulated dietary supplement (Incoxil®, Fempharma, LLC, Debrecen, Hungary) (treatment group) or placebo (control group). Women in the treatment group were asked to take the oral dietary supplement every day for six weeks. Dietary supplement of the treatment group included creatine monohydrate 3 g/day, leucine 0.5 g/day, zinc sulfate heptahydrate 5 mg/day, calcium citrate 120 mg/day, and magnesium citrate 60 mg/day) as a water-soluble powder. While in the control group, women received daily supplementation with maltodextrin as a water-soluble powder. The color, texture, solubility, taste, and volume of the powder were similar between the two groups.

After randomization, subjects received the dietary supplement in a sealed, uniform box. The completely identical boxes were labeled with codes. The codes were not known by the researchers participating in the clinical trial. The code could not identify the patients. Participants did not know what kind of preparations they were given; they were assigned randomly without informing them which group they belonged to.

After enrollment based on the MESA questionnaire, women were asked to complete the Urinary Distress Inventory (UDI-6) and Incontinence Impact Questionnaire (IIQ-7). Standardized pelvic exam, including POP-Q measurement, Oxford scale assessment of the pelvic floor strength, the strength of voluntary contractions of vaginal muscles was measured by a perineometer device (Peritron, Laborie, Williston, Vermont, USA) in a standardized fashion [25, 26] and the biomechanical integrity of the pelvic floor was evaluated by VTI [27]. Patients received detailed pelvic floor muscle exercise instruction and training with the aid of the perineometer. Study participants were instructed to perform daily pelvic floor muscle exercises at an intensity of at least 65–75% of one repetition maximum of 45 pelvic floor muscle (PFM) contractions per day (15 PFM contractions performed three times per day) for six weeks as per previous protocols [7, 8].

At the six weeks visit, study participants were asked to complete the UDI-6 and IIQ-7 questionnaires again. In addition, repeat pelvic exams, including POP-Q measurement, Oxford scale assessment of the pelvic floor strength, the strength of voluntary contractions of vaginal muscles was measured by a perineometer device in a standardized fashion, and VTI evaluated the biomechanical integrity of the pelvic floor.

The primary outcome was the UDI-6 score. Secondary outcomes were the Incontinence Impact Questionnaire (IIQ-7) score, Patient’s Global Impression of Severity (PGI‐S) and Patient’s Global Impression of Improvement (PGI‐I), Biomechanical Integrity score as measured by the Vaginal Tactile Imager and vaginal squeeze pressure measured by perineometer.

### Questionnaires

#### Medical, epidemiologic, and social aspects of aging (MESA) questionnaire

The MESA questionnaire is a reliable and validated tool developed and validated to identify the urgency- or stress-predominant component of MUI and assess the severity of symptoms [23, 24]. The MESA questionnaire consists of two separate parts, nine questions regarding SUI and six questions concerning urgency urinary incontinence (UUI). The maximum total score for SUI is twenty-seven, while eighteen for UUI, with 0 to 3 allocated to the answers of never/rarely/sometimes/often. The stress/urge index is calculated to detect stress- or urge-predominant MUI. The indexes are obtained by dividing the actual score of each category by the maximum total possible. The MUI is categorized as stress-predominant when the stress index is greater than the urge index. To assess the severity of stress incontinence, the total score of stress (maximum = 27) is divided into three third categories. For the stress category, scores of 1 to 9 are assigned as mild, 10 to 18 as moderate, and 13 to 18 as severe.

#### Urogenital distress inventory (UDI-6) and incontinence impact questionnaire (IIQ-7)

UDI-6 is a short version of a condition-specific quality-of-life instrument [28, 29]. UDI-6 consists of six items. To specify the severity of urinary distress symptoms, patients’ response options ranged from 0 (“no symptoms”) to 4 (“quite a bit”). To calculate the score, the mean score of answered items within each component is multiplied by 25 to obtain the scale score (range 0–100). Higher scores in UDI-6 indicate a higher disability. IIQ-7 is a urinary incontinence-specific psychometric questionnaire. This questionnaire assesses the psychosocial impact of UI in women. It consists of 7 items. The total score ranges from 0 to 100 [28, 29]. Higher score indicates a worse quality of life.

#### Patient global impression of severity and improvement question (PGI-S and PGI-I)

The Patient Global Impression of Severity (PGI-S) is a global index that may be used to rate the severity of a specific condition (a single-state scale). The PGI-S was validated on women with stress urinary incontinence [30]. PGI-S, range 0 = no symptoms to 4 = severe symptoms, MID = 1 [31]. The PGI-I is a seven-point transition scale that comprises a single question asking patients to rate their urinary tract condition now as compared with how it was before treatment on a scale from 1 (very much better) to 7 (very much worse) [30].

#### Vaginal tactile imager – biomechanical integrity score [27]

We used Vaginal Tactile Imager (VTI) model 2 S for biomechanical mapping of the pelvic floor. Besides 96 pressure (tactile) sensors aligned on both sides of the probe, an orientation sensor and temperature controller have the equipment’s probe. Depending on the setting, the probe acquires pressure responses from the anterior and posterior or left and right vaginal walls. As a result, the images integrate all the acquired pressure and positioning data for each pressure-sensing. The VTI examination procedure consists of eight Tests as follows: (1) probe insertion, (2) elevation, (3) rotation, (4) Valsalva maneuver, (5) voluntary muscle contraction (anterior versus posterior compartments), (6) voluntary muscle contraction (left versus right side), (7) involuntary relaxation, and (8) reflex muscle contraction (cough). Tests 1, 2, 4, 5, 7, and 8 provide data for anterior/posterior compartments; Test 3 provides data for 360 degrees; Test 6 provides data for left/right sides (see Table 1). This VTI probe allows 3–15 mm tissue deformation at the probe insertion (Test 1), 20–45 mm tissue deformation at the probe elevation (Test 2), and 5–7 mm deformation at the probe rotation (Test 3) and recording of dynamic responses at Valsalva maneuver, pelvic muscle contractions, and relaxation (Tests 4–8). The probe maneuvers in Tests 1–3 are used to accumulate multiple pressure patterns from the tissue surface and create an integrated tactile image for the investigated area employing the image composition algorithms. The spatial gradients ∂P(x, y)/∂y for anterior and posterior compartments are calculated within the acquired tactile images in Test 1 and 2; the y-coordinate is directed orthogonally from the vaginal channel coming through anterior-posterior compartments, and the x-coordinate is located on the vaginal channel. The VTI software calculates (automatically) 52 VTI parameters for eight test procedures listed in Table 1. BI-score covers biomechanical aspects of the pelvic floor, which include tissue elasticity, pelvic support, muscle contraction, involuntary relaxation, and mobility.

**Table 1:** Demographics and Clinical Characteristics

	Control Group (N = 16)	Treatment Group (N = 16)	P-value
Age (years, mean ± SD	57 ± 12	53 ± 10	0.28
Gravida (median, range)	2 (1–4)	2 (1–3)	NS
Parity (median, range)	2 (1–3)	2 (1–5)	NS
BMI (kg/m^2^, mean ± SD)	26.6 ± 5.3	28.6 ± 5.9	0.32
History of vaginal delivery (n, %)	14 (87)	16 (100)	NS
Postmenopausal (n, %)	9 (56)	8 (50)	NS
POP-Q stage (median, range)	2 (1–2)	2 (1–2)	NS
MESA SUI index (%, mean ± SD)	57 ± 20	49 ± 19	0.25
MESA UUI index (%, mean ± SD)	13 ± 14	17 ± 13	0.46
MESA SUI score (mean ± SD)	15.3 ± 5.5	13.2 ± 5.0	0.25
Severity of SUI			
Mild (n, %)	3 (19)	5 (31)	
Moderate (n, %)	8 (50)	8 (50)	
Severe (n, %)	5 (31)	3 (19)	

NS: not significant, POP-Q: pelvic organ prolapse quantification, SUI: stress urinary incontinence, UUI: urge urinary incontinence, MESA: Medical, epidemiologic, and social aspects of aging urinary incontinence questionnaire, Stress urinary incontinence severity category: scores of 1 to 9 is assigned as mild, 10 to 18 as moderate, and 19 to 27 as severe

### Statistics

For statistical calculations, we used SigmaStat/SPSS (Systat Software Inc., San Jose, CA, USA) software. Descriptive statistics were calculated for all variables of interest. Means and standard deviations were calculated for continuous outcomes. Frequency and percentage were calculated for categorical outcomes. The Fisher exact test was used to compare frequencies. Student’s t-test was used to compare the mean values between the two groups. Paired t-test was used to compare paired data, which was obtained at baseline and six weeks. Wilcoxon signed rank test was used to compare the VTI data. The study has adequate power to detect a difference after 32 patients’ enrollment. Power analysis was performed [G*Power Statistical Power Analyses Software [32]] based on our pilot study, which revealed that in our population of women with SUI, the baseline UDI score was 36 ± 15. To have a power of 80% and a significance level of 5% with a large effect size (Cohen’s d = 1.06) to detect a decrease of 16 points in the UDI-6 score, a sample size of 32 was needed with 16 patients in each arm of our RCT. The MCID for the UDI-6 questionnaire is 11 points [33]. Calculating with a dropout rate of 10%, thirty-four women were needed to be enrolled. Statistical significance was defined as a P-value < 0.05 using two-tailed tests.

## Results

**Fig. 1:**
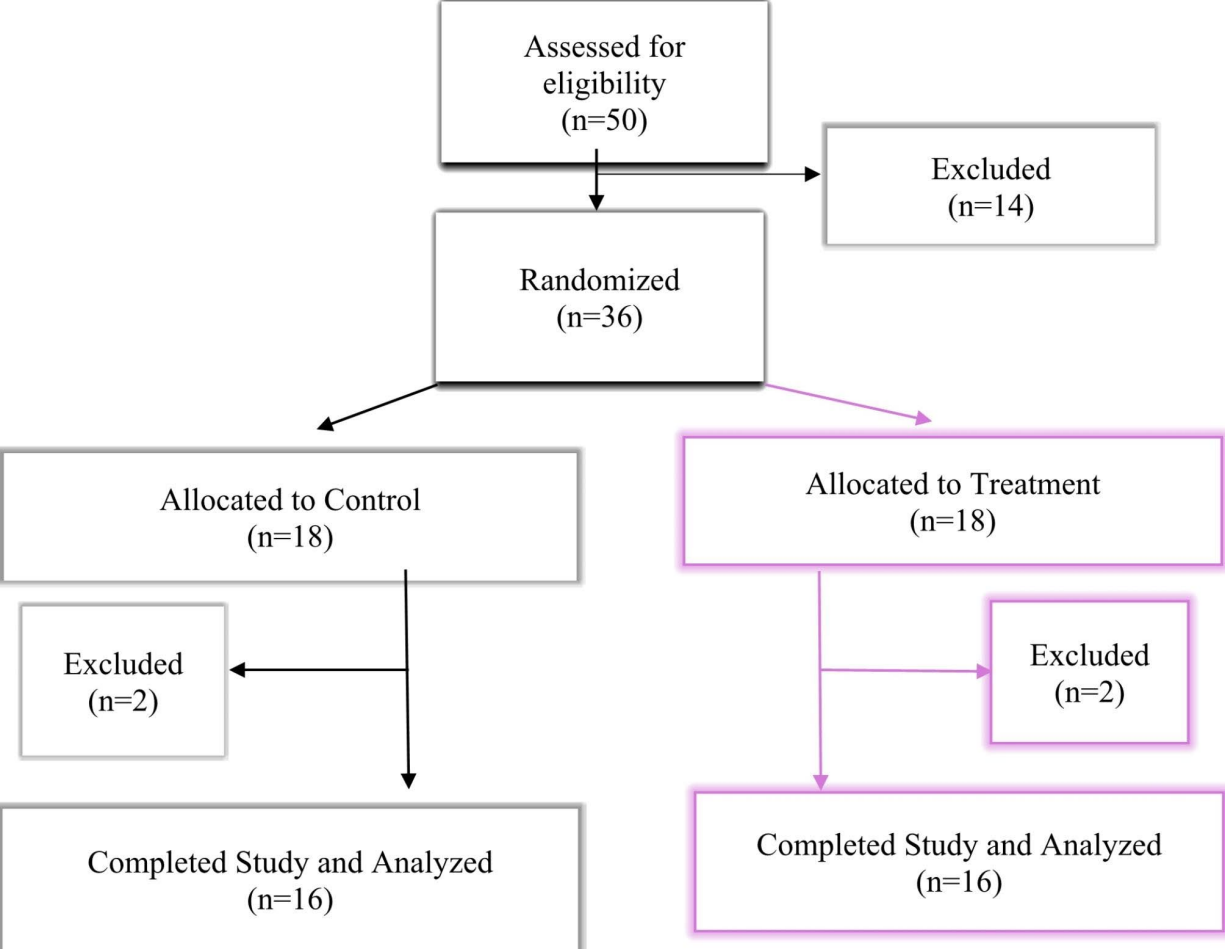
The flow of participants through the randomized clinical trial

Figure 1 summarizes the assessment for eligibility, randomization, allocation, and exclusions of participants. Of the fifty women screened, thirty-six met the entry criteria and were randomized. Eighteen women were randomly assigned to the control group and eighteen to the treatment group. Thirty-two women completed the trial. Of the fourteen excluded participants, ten did not meet the inclusion criteria, four decided not to participate in the trial.

Of the eighteen individuals randomly assigned to the treatment group, two did not complete the trial: one was lost to follow-up, and one withdrew from participation because of lack of time. Of the eighteen women randomized to the control group, two did not complete the trial: one was lost to follow-up, and one withdrew informed consent before taking any supplement. In total, thirty-six women completed the trial: sixteen in the control group and sixteen in the treatment group.

There were no demographic differences between the two groups (Table 1). MESA stress urinary incontinence (SUI) scores were similar between the control and treatment groups (mean ± SD, 15.3 ± 5.5 vs. 13.2 ± 5.0; P = 0.25). Between-group analysis revealed no significant differences between the control and treatment group except for mean change (increase) in vaginal squeeze pressure [(cmH2O, mean ± SD), 5 ± 12 vs. 15 ± 15, P = 0.04] and mean change (decrease) in PGI-S score [(mean ± SD), -0.2 ± 0.9 vs. -0.8 ± 0.8, P = 0.04] (Table 2). Out of the 52 examined VTI parameters, significantly more parameters improved in the treatment group compared to the control group (11/52 vs. 3/52, P = 0.04) (Tables 3 and 4).

**Table 2:** Primary and secondary outcomes at baseline and completion of the study. Between-group analysis was performed

	Control Group (N = 16)	Treatment Group (N = 16)	P-value
UDI-6 score before (mean ± SD)	43 ± 18	45 ± 21	0.77
UDI-6 score after (mean ± SD)	33 ± 26	29 ± 22	0.69
UDI-6 score change (mean ± SD)	-10 ± 31	-**16** ± 25	0.58
IIQ-7 score before (mean ± SD)	48 ± 23	50 ± 30	0.76
IIQ-7 score after (mean ± SD)	40 ± 28	30 ± 21	0.31
IIQ-7 score change (mean ± SD)	-8 ± 30	-20 ± 27	0.25
Maximum vaginal squeeze pressure before (cmH_2_O, mean ± SD)	36 ± 18	30 ± 15	0.27
Maximum vaginal squeeze pressure after (cmH_2_O)	41 ± 21	45 ± 28	0.71
Change in vaginal squeeze pressure (cmH_2_O, mean ± SD)	5 ± 12	15 ± 15	**0.04**
Oxford Scale before (mean ± SD)	2.4 ± 0.8	2.2 ± 0.6	0.51
Oxford Scale after (mean ± SD)	2.9 ± 0.5	2.8 ± 0.5	0.53
Oxford Scale change (mean ± SD)	0.5 ± 0.7	0.6 ± 0.5	0.78
PGI-S score before (mean ± SD)	2.8 ± 0.8	3.1 ± 0.8	0.31
PGI-S score after (mean ± SD)	2.6 ± 0.8	2.3 ± 0.8	0.19
PGI-S score change (mean ± SD)	-0.2 ± 0.9	-0.8 ± 0.8	**0.04**
PGI-I score (mean ± SD)	2.4 ± 1.0	2.6 ± 0.9	0.61

Urogenital Distress Inventory (UDI-6), Incontinence Impact Questionnaire (IIQ-7), Patient Global Impression of Severity and Improvement question (PGI-S and PGI-I)

**Table 3:** Fifty-two VTI parameters for women in the control group at baseline and completion of trial

VTI Parameter No.	Parameter units	Average (Control Group at Baseline) (N = 16)	Average (Control roup at 6 weeks) (N = 16)	100* (Post-Pre)/Pre, %	Pre-treatment standard deviation	Post-treatment standard deviation	P-value for Wilcoxon signed rank test
1	N	0.55	0.67	20.8	0.42	0.40	0.4545
2	mJ	21.38	26.49	23.9	13.84	17.09	0.0768
3	kPa/mm	0.83	1.04	25.0	0.67	0.81	0.3018
4	kPa/mm	0.64	0.80	25.8	0.49	0.53	0.4545
5	kPa	14.38	16.49	14.7	11.47	10.98	0.4545
6	kPa	9.13	11.76	28.8	5.00	6.69	0.0042
7	kPa	7.69	9.04	17.5	7.54	9.06	0.8036
8	kPa	5.44	6.13	12.6	3.42	4.46	1.0000
9	kPa	4.40	5.39	22.4	3.09	3.08	0.8036
10	kPa	5.58	7.13	27.8	3.72	4.35	0.0213
11	kPa	6.51	6.19	-4.9	3.56	3.50	1.0000
12	kPa	4.83	4.57	-5.3	2.95	3.88	0.6072
13	kPa/mm	0.69	0.58	-15.1	1.18	1.15	1.0000
14	kPa/mm	0.33	0.31	-5.7	0.41	0.31	1.0000
15	kPa/mm	0.20	0.26	26.2	0.18	0.18	0.4545
16	kPa/mm	0.60	0.47	-22.1	1.03	0.30	0.8036
17	kPa/mm	0.34	0.25	-25.8	0.46	0.14	0.8036
18	kPa/mm	0.23	0.19	-14.7	0.19	0.17	1.0000
19	kPa	11.95	15.59	30.4	8.47	9.92	0.2101
20	N	1.91	2.45	28.2	0.88	1.24	0.4545
21	N	0.98	1.17	18.9	0.51	0.79	0.6072
22	kPa	3.95	4.74	20.1	1.75	2.68	0.2101
23	kPa	4.05	4.38	8.2	2.34	3.47	0.4545
24	kPa	4.59	5.68	23.8	2.38	3.42	1.0000
25	N	0.99	0.96	-2.6	0.72	0.48	1.0000
26	kPa	7.25	5.31	-26.7	8.17	3.18	0.8036
27	mm	1.27	2.44	92.6	6.90	5.38	1.0000
28	N	0.96	1.09	13.8	0.66	0.59	0.8036
29	kPa	4.90	5.40	10.2	3.33	3.88	1.0000
30	mm	-0.08	2.28	-2900.0	4.04	6.10	0.8036
31	N	1.17	1.50	27.8	0.83	0.95	0.8036
32	kPa	17.55	21.74	23.9	16.65	17.57	1.0000
33	kPa	23.21	27.67	19.2	17.84	19.87	0.4545
34	N	1.25	1.68	35.2	0.65	0.78	0.4545
35	kPa	9.49	12.01	26.5	6.09	6.54	0.2101
36	kPa	14.54	17.81	22.4	7.36	9.19	0.0768
37	N	0.58	0.79	36.8	0.39	0.55	0.4545
38	kPa	4.73	5.59	18.2	3.86	4.34	0.4240
39	kPa	8.40	9.41	12.0	5.30	6.63	0.6072
40	N	0.54	0.76	41.6	0.36	0.56	0.8036
41	kPa	4.54	5.28	16.2	3.78	4.60	0.2101
42	kPa	7.99	9.15	14.5	5.51	6.75	0.1796
43	kPa/s	-1.82	-0.71	-61.0	1.84	2.21	0.6072
44	%/s	-6.36	-2.87	-54.9	6.94	9.32	0.2101
45	kPa/s	-1.18	-0.95	-19.6	1.16	1.10	1.0000
46	%/s	-7.11	-4.84	-31.8	5.04	6.60	0.3018
47	N	1.40	1.65	17.5	0.81	0.78	0.4545
48	kPa	8.23	8.45	2.7	4.71	5.05	0.8036
49	mm	8.69	2.47	-71.6	7.24	4.88	0.0042
50	N	1.51	1.85	23.0	0.99	0.82	0.2101
51	kPa	8.16	9.49	16.3	4.46	4.86	0.4545
52	mm	6.05	4.24	-29.9	5.68	4.61	0.4240

**Table 4:** Fifty-two VTI parameters for women in the treatment group at baseline and completion of trial

VTI parameter No.	Parameter units	Average (Treatment Group at baselined) (N = 16)	Average (Treatment Group at 6 (weeks) (N = 16)	100 (Post-Pre)/Pre, %	Pre-treatment standard deviation	Post-treatment standard deviation	P-value for Wilcoxon signed rank test
1	N	0.41	0.54	33.2	0.21	0.23	0.0111
2	mJ	17.08	20.93	22.5	8.29	9.81	0.1189
3	kPa/mm	0.49	0.60	21.7	0.37	0.51	0.3336
4	kPa/mm	0.35	0.45	27.0	0.20	0.28	0.4212
5	kPa	8.02	10.81	34.8	4.67	5.34	0.0497
6	kPa	7.46	8.51	14.0	4.78	4.09	0.1635
7	kPa	5.11	5.39	5.5	2.71	2.80	0.8581
8	kPa	4.23	4.93	16.4	2.20	2.75	0.1470
9	kPa	4.90	4.94	0.8	3.54	3.74	0.9875
10	kPa	3.48	4.49	29.1	1.41	1.78	0.0659
11	kPa	4.95	5.43	9.7	2.38	1.62	0.1925
12	kPa	4.29	4.17	-2.8	2.88	2.16	0.7734
13	kPa/mm	0.23	0.34	50.7	0.20	0.28	0.1238
14	kPa/mm	0.15	0.19	24.6	0.14	0.14	0.3656
15	kPa/mm	0.17	0.29	63.4	0.14	0.51	0.9233
16	kPa/mm	0.20	0.34	71.7	0.08	0.36	0.1152
17	kPa/mm	0.20	0.18	-8.6	0.14	0.11	0.7542
18	kPa/mm	0.21	0.19	-9.7	0.17	0.16	0.4294
19	kPa	7.07	10.24	44.9	1.33	3.48	0.0016
20	N	1.53	1.79	16.9	0.42	0.58	0.1923
21	N	0.92	1.08	17.7	0.43	0.49	0.2025
22	kPa	3.81	4.15	8.9	1.65	2.45	0.7020
23	kPa	3.45	4.81	39.4	1.46	2.65	0.1101
24	kPa	4.52	4.92	8.8	1.78	2.43	0.3494
25	N	1.22	1.26	3.2	0.64	0.47	0.8469
26	kPa	6.80	7.15	5.2	4.10	4.89	0.5900
27	mm	2.26	1.62	-28.3	5.18	4.85	0.7720
28	N	1.23	1.25	1.5	0.65	0.53	0.5614
29	kPa	5.81	5.91	1.7	2.87	2.85	0.5708
30	mm	1.16	2.51	116.7	4.33	4.39	0.6785
31	N	1.00	1.09	9.6	0.58	0.72	0.5245
32	kPa	10.72	10.27	-4.2	10.30	11.03	0.9780
33	kPa	14.83	14.70	-0.9	10.86	12.67	0.6483
34	N	1.12	1.24	10.8	0.68	0.66	0.4353
35	kPa	7.92	7.98	0.8	5.57	4.68	1.0000
36	kPa	11.75	12.19	3.7	6.22	5.28	0.6788
37	N	0.55	0.78	43.0	0.49	0.56	0.0125
38	kPa	3.74	4.60	23.0	4.26	4.04	0.0835
39	kPa	7.39	7.51	1.5	6.32	4.96	0.7516
40	N	0.51	0.81	59.5	0.48	0.65	0.0203
41	kPa	3.83	5.28	38.0	4.54	5.34	0.0479
42	kPa	7.19	8.31	15.5	6.65	7.10	0.0497
43	kPa/s	-1.66	-0.44	-73.4	2.32	1.00	0.0033
44	%/s	-11.30	-4.04	-64.3	5.88	5.09	0.0007
45	kPa/s	-1.03	-0.35	-65.6	0.95	0.43	0.0043
46	%/s	-10.97	-3.49	-68.2	7.53	3.39	0.0004
47	N	1.95	1.85	-4.9	0.74	0.79	0.7615
48	kPa	10.01	9.07	-9.5	3.29	3.01	0.5702
49	mm	9.35	4.15	-55.7	8.29	6.53	0.1311
50	N	2.04	1.98	-3.0	0.78	0.93	0.9891
51	kPa	9.32	8.26	-11.4	3.77	2.82	0.2348
52	mm	7.53	6.26	-16.8	6.85	7.18	0.6286

Within-group analysis showed that UDI-6 and IIQ-7 scores improved significantly from baseline to six weeks in the treatment group but not in the control group [UDI-6 score (mean ± SD) 45 ± 21 vs. 29 ± 21, P = 0.02 (treatment group), 43 ± 18 vs. 33 ± 26, P = 0.22 (control group)] [IIQ-7 score before (mean ± SD) 50 ± 30 vs. 30 ± 21, P = 0.01 (treatment group) 48 ± 23 vs.40 ± 28, P = 0.36 (control group)] (Table 5). Similarly, maximum vaginal squeeze pressure was significantly stronger in the treatment group compared to baseline after six weeks but not in the control group [Maximum vaginal squeeze pressure (cmH2O, mean ± SD), 30 ± 15 vs. 45 ± 28, P = 0.001 (treatment group) and 36 ± 18 vs. 41 ± 21, P = 0.13 (control group)]. PGI-S scores only improved in the treatment group from baseline to six weeks after treatment [PGI-S score (mean ± SD) 3.1 ± 0.8 vs. 2.3 ± 0.8, P = 0.0001].

**Table 5:** Primary and secondary outcomes at baseline and the completion of study. Within-group analysis was performed

	Control Group at Baseline (N = 16)	Control Group at 6 weeks (N = 16)	P-value	Treatment Group at Baseline (N = 16)	Treatment Group at 6 weeks (N = 16)	P-value
UDI-6 score (mean ± SD)	43 ± 18	33 ± 26	0.22	45 ± 21	29 ± 21	**0.02**
IIQ-7 score before (mean ± SD)	48 ± 23	40 ± 28	0.36	50 ± 30	30 ± 21	**0.01**
Maximum vaginal squeeze pressure (cmH_2_O, mean ± SD)	36 ± 18	41 ± 21	0.13	30 ± 15	45 ± 28	**0.001**
Oxford Scale (mean ± SD)	2.4 ± 0.8	2.9 ± 0.5	**0.01**	2.2 ± 0.6	2.8 ± 0.5	**0.0005**
PGI-S score (mean ± SD)	2.8 ± 0.8	2.6 ± 0.8	0.45	3.1 ± 0.8	2.3 ± 0.8	**0.0001**

Urogenital Distress Inventory (UDI-6), Incontinence Impact Questionnaire (IIQ-7), Patient Global Impression of Severity (PGI-S)

**Fig. 2:**
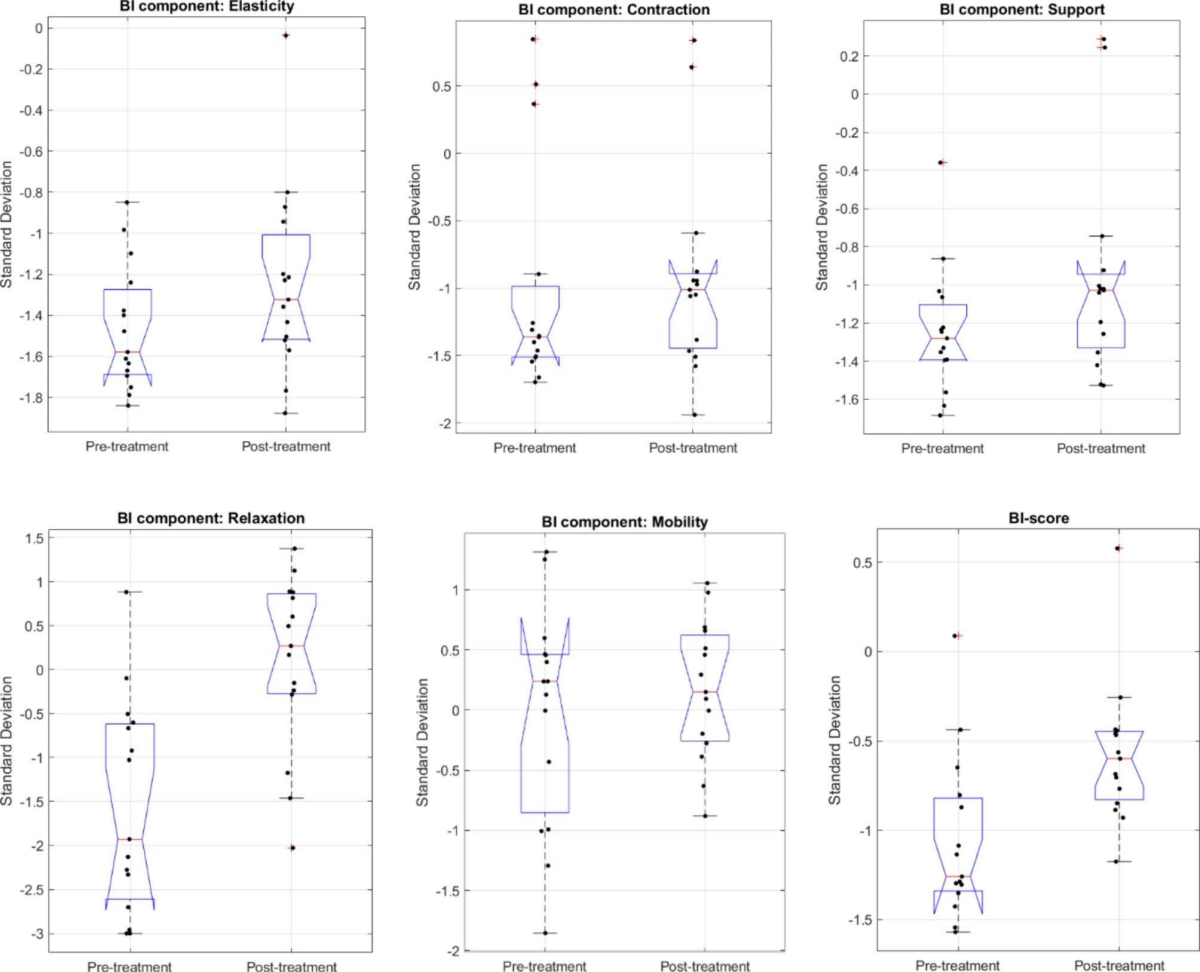
Boxplots for components of the Biomechanical Integrity Score At baseline and at the completion of the study: Elasticity (P = 0.035), Contraction, Support, Relaxation (P = 0.0004), and Mobility. BI-score was significantly improved (P = 0.0012)

Vaginal Tactile Imaging revealed that in the control group, only three VTI parameters improved (VTI #6, 10, 49), while in the treatment group, eleven parameters were better (VTI #1,5, 19, 37, 40–46) (Tables 3 and 4). None of the VTI parameters worsened during the trial (Tables 3 and 4). BI-score, on average, improved significantly in the treatment (Fig. 2) and control group as well [SD unit, mean, from − 1.06 to -0.58, P = 0.001 (treatment group) and from − 0.66 to -0.42, P = 0.04 (control group)] (Tables 6 and 7). Nevertheless, women in the treatment, on average, improved twice as much in their BI-score as women in the control group (-0.49 vs. -0.24). There was a significant improvement in elasticity and relaxation in the treatment group, while in the control group, only in contraction (Tables 6 and 7).

**Table 6:** Biomechanical Integrity Score (BI-score) in Control Group

	Parameter units	Control Group at Baseline (N = 16)	Control Group at 6 weeks (N = 16)	*P*-value for Wilcoxon signed rank test
Elasticity	SD	-1.24	-1.02	0.0787
Support	SD	-0.74	-0.70	0.4380
**Contraction**	**SD**	**-0.87**	**-0.55**	**0.0084**
Relaxation	SD	-0.68	-0.06	0.0703
Mobility	SD	0.23	0.22	0.3520
**BI-score**	**SD**	**-0.66**	**-0.42**	**0.0494**

BI-score is measured by Vaginal Tactile Imager at baseline and the completion of the study

**Table 7:** Biomechanical Integrity Score (BI-score) in Treatment Group

	Parameter units	Treatment Group at Baseline (N = 16)	Treatment Group at 6 weeks (N = 16)	*P*-value for Wilcoxon signed rank test
**Elasticity**	**SD**	**-1.47**	**-1.24**	**0.0353**
Support	SD	-1.24	-0.97	0.1070
Contraction	SD	-1.02	-0.92	0.1876
**Relaxation**	**SD**	**-1.55**	**0.09**	**0.0004**
Mobility	SD	-0.03	0.17	0.5995
**BI-score**	**SD**	**-1.06**	**-0.58**	**0.0012**

BI-score is measured by Vaginal Tactile Imager at baseline and the completion of the study

## Discussion

We are the first to report the effects of a specially formulated supplement on urinary incontinence symptoms while study participants were engaged in daily PFMT. We have found that daily supplementation, in addition to regular pelvic floor muscle exercise at an intensity of at least 65–75% of one repetition maximum of 45 pelvic floor muscle (PFM) contractions per day (15 PFM contractions performed three times per day) for six weeks resulted in greater vaginal muscle strength change and larger change (decrease) in the PGI-S of UI and improved biomechanical integrity of the pelvic floor (BI-score).

Most professional guidelines recommend conservative treatment for first-line treatment of SUI. The European Association of Urology (EAU) recommends that providers should offer supervised intensive pelvic floor muscle training (PFMT), lasting at least three months, as first-line therapy to all women with SUI. At the same time, the American College of Obstetricians and Gynecologists (ACOG) states that behavioral modification and pelvic floor muscle exercises improve symptoms of urinary incontinence and may be recommended as an initial, noninvasive treatment in any women [6]. Although PFMT is widely used, there is no consensus on the number, duration, and intensity of the exercises. In our trial, we used a program similar to the ESTEEM trial’s [7, 8]. This specific PFMT volume has been shown to be manageable with regard to exercise adherence while improving UI symptoms [10, 11]. A recent Cochrane systemic review compared PFMT with no treatment and found confidence that PFMT can cure or improve symptoms of SUI. Women with SUI were eight times more likely to report cure in the PFMT group [12]. In addition, vaginal squeeze pressure has been used as a marker for pelvic floor function [34]. PFMT has been shown to increase vaginal squeeze pressure and improve UI symptoms [35, 36]. We have seen a greater increase in vaginal squeeze pressure in the treatment group, likely contributing to the improved PGI-S change.

The number of publications examining the relationship between nutrition and PFMT is very small and almost exclusively focused on the role of nutrition in obesity [37]. We believe that our specially formulated supplement contributed to the improvement of the pelvic floor muscles secondary to the unique composition of the supplement. Our key ingredients were creatine, leucine, zinc, calcium, and magnesium. Evidence supports that creatine supplementation may improve health status as individuals age and increase strength and/or muscle mass [38]. Increased vaginal-pelvic muscle strength and activity may result in better urinary function, especially in women with SUI. Previous studies have shown that even long-term supplementation with creatine (up to 30 g/day for five years) is safe and well-tolerated in healthy individuals and the elderly as well [17]. In addition to creatine, leucine was one of the key ingredients of the supplement. It is directly involved in protein synthesis as it directly stimulates protein synthesis in muscles, but it has never been shown before to contribute to pelvic floor muscle strength [19–21]. Leucine can also prevent muscular weakness related to aging, and research has shown leucine’s beneficial role in enhancing muscle protein synthesis [19–21]. The third key component of the stu-sied food supplement is zinc. It is essential in connective tissue biosynthesis and homeostasis [39]. Zinc levels have been shown to decline with age [40], and therapeutic interventions aiming to treat vaginal atrophy have been shown to increase the zinc levels in the vagina [41]. In addition, calcium and magnesium are critical elements in muscle function to maintain physical fitness [42]. Altered calcium homeostasis, as frequently found in the elderly, together with oxidative stress, is implicated in augmented muscle damage, decreased muscle mass, and impaired muscle contractility [42]. On the other hand, magnesium is involved in protein and ATP synthesis and is responsible for muscle relaxation [42]. A randomized clinical trial revealed that elderly women receiving daily supplements with magnesium for 12 weeks, in addition to an exercise program, had improved physical performance, suggesting a role for magnesium supplementation in maintaining muscle function and helping to delay sarcopenia [43]. Our findings indicate that the unique composition of our supplement containing all key ingredients (creatine, leucine, zinc, magnesium, and calcium) in a unique ratio may be, in part, responsible for the observed beneficial effects on pelvic floor muscle strength and UI symptoms.

Vaginal Tactile Imaging revealed that only three VTI parameters improved in the control group. In contrast, eleven parameters were better in the treatment group, and none of the VTI parameters got worse during the trial. These findings are reassuring and consistent with our clinical findings that women in the treatment group had a larger improvement in UI symptoms, similarly to the improvement in the biomechanical integrity of the pelvic floor. Women in the treatment, on average, improved twice as much in their BI-score as women in the control group. It is not entirely understood how PFMT improves UI symptoms but likely through the improvement of the integrity and coordination of the pelvic floor muscles. In the treatment group, there was a significant improvement in elasticity and relaxation, while in the control group, only in contraction, consistent with the likely impact of PFMT through improving muscle function.

The strength of this study is its randomized nature and the first trial testing the effects of a food supplement in combination with PFMT on women with stress pre-dominant UI. An important strength of the study was that participants were blinded to the allocation. Another strength of this RCT is that the evaluators performing the perinoemetry, the vaginal tactile imaging, and the pelvic floor assessment were also blinded to the allocation. In addition, the objective measurements of pelvic floor integrity by VTI were complemented by subjective patient assessments based on validated questionaries. Also, a strength of this study was our participants’ high compliance with taking the supplements and adhering to the daily PFMT based on self-reporting (~ 90%). The trial discontinuation rate in the treatment group was identical in the control and treatment groups. The high (~ 90%) attrition rate was another strength of this randomized controlled trial.

One of the main weaknesses of our study was the lack of power to detect differences in several of the secondary outcomes; overall, a relatively low number of women have been enrolled. In addition, we have assumed a very large effect size (Cohen’s d = 1.06) which is difficult to achieve in 6 weeks. A weakness of the study was that we did not assess urinary incontinence by urodynamics or pad-test but only with validated questionaries. Also, a weakness of our study was the homogenous nondiverse study population, which can be explained by the fact that the vast majority of the local population belongs to only one race and ethnicity. Finally, our trial does not provide an accurate insight into the supplement’s mechanism of action. It is possible that the observed effects are secondary through generalized improvement in muscle strength rather than a direct effect on pelvic floor muscles and extracellular matrix.

## Conclusions

In summary, although we did not find a significant difference in urinary symptoms between the treatment group and the placebo group at six weeks at the proposed large effect size (Cohen’s d = 1.06), there was a significant improvement in the treatment group in all examined primary and secondary outcomes compared to baseline. However, no difference was found in the control group. In our trial, short-term (six weeks) pelvic floor muscle exercise did not improve significantly urinary incontinence symptoms. This finding is not a surprise since most guidelines recommend twelve weeks of PFMT for achieving improvement in SUI symptoms. However, we are the first to report that when daily PFMT was augmented by a specially formulated supplement, the urinary incontinence symptoms, biomechanical integrity of the pelvic floor, and pelvic floor muscle strength significantly improved in a relatively short time period. Based on our findings, we suspect that the daily supplementation with Incoxil®, while pelvic floor training was ongoing, resulted in faster improvement in pelvic floor muscle strength and biomechanical integrity, which indeed led to better urinary control after only six weeks of treatment. Our findings are preliminary, and additional studies with a larger sample size are needed to verify our hypothesis.

## Data Availability

The datasets used and/or analyzed during the current study are available from the corresponding author on reasonable request.

## List of Abbreviations

UI: Urinary incontinence
SUI: Stress urinary incontinence
MUI: Mixed urinary incontinence
PFMT: Pelvic floor muscle training
PFM: Pelvic floor muscle
MESA: Medical, Epidemiologic, and Social Aspects of Aging questionnaire
UDI-6: Urogenital Distress Inventory Short Form
IIQ-7: Incontinence Impact Questionnaire
PGI-S: Patient’s Global Impression of Severity
PGI-I: Patient’s Global Impression of Improvement
BI-score: Biomechanical Integrity score
VTI: Vaginal Tactile Imager
EFSA: European Food Safety Authority
SD: Standard deviation
